# Redetermination of di­aqua­tris­(4-oxo­pent-2-en-2-olato-κ^2^ *O*,*O*′)lanthanum(III)

**DOI:** 10.1107/S1600536814013336

**Published:** 2014-06-14

**Authors:** Toru Okawara, Kohei Ishihama, Kenji Takehara

**Affiliations:** a Department of Materials Science and Chemical Engineering, Kitakyushu National College of Technology, Shi-i 5-20-1, Kokuraminami-ku, Kitakyushu, Fukuoka 802-0985, Japan

## Abstract

The structure of the title compound, [La(C_5_H_7_O_2_)_3_(H_2_O)_2_], has been redetermined to modern standards with anisotropic displacement parameters for all non-H atoms and the hydrogen-bonding pattern unambiguously established [for the previous study, see Phillips *et al.* (1968 ▶). *Inorg. Chem.* **7**, 2295–2299]. The La^3+^ ion is coordinated by three *O*,*O*′-bidentate acetyl­acetate (acac^−^) ligands and two water mol­ecules, resulting in a fairly regular square-anti­prismatic LaO_8_ coordination geometry, with both aqua ligands part of the same square face. In the crystal, the neutral complex mol­ecules are linked into [110] chains by O—H⋯O hydrogen bonds.

## Related literature   

For the previous report on the title compound, see: Phillips *et al.* (1968 ▶). For related tris­(acetyl­acetonato)lanthanide complexes, see: Watkins *et al.* (1969 ▶); Kooijman *et al.* (2000 ▶). For other lanthanide complexes, see: Richardson *et al.* (1968 ▶); Lama *et al.* (2007 ▶).
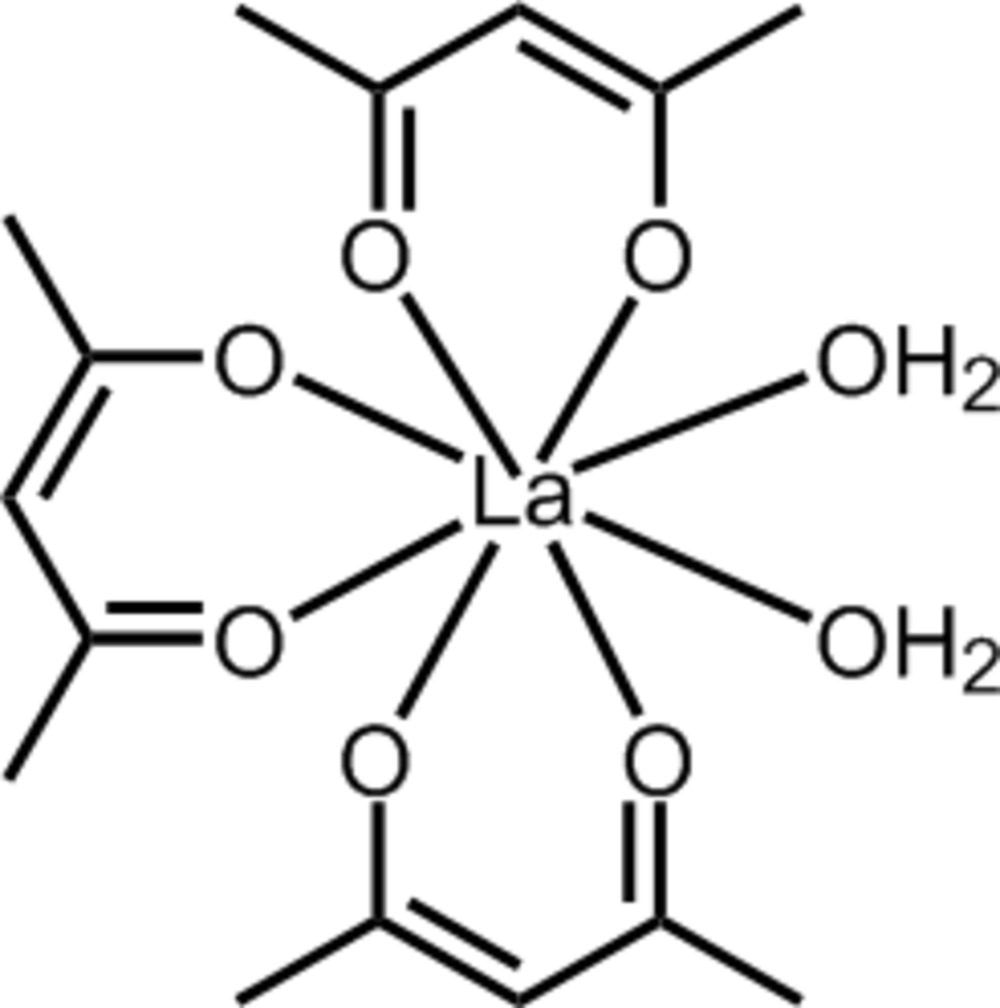


## Experimental   

### 

#### Crystal data  

[La(C_5_H_7_O_2_)_3_(H_2_O)_2_]
*M* *_r_* = 472.26
Triclinic, 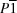

*a* = 8.9245 (12) Å
*b* = 10.6597 (15) Å
*c* = 11.3727 (15) Å
α = 96.614 (2)°
β = 100.601 (2)°
γ = 114.325 (2)°
*V* = 946.8 (2) Å^3^
*Z* = 2
Mo *K*α radiation
μ = 2.29 mm^−1^
*T* = 100 K
0.50 × 0.50 × 0.22 mm

#### Data collection  

Bruker APEXII CCD diffractometer
Absorption correction: multi-scan (*SADABS*; Bruker, 2008 ▶) *T*_min_ = 0.40, *T*_max_ = 0.63
13810 measured reflections
5213 independent reflections
5068 reflections with *I* > 2σ(*I*)
*R*_int_ = 0.030

#### Refinement  

*R*[*F* ^2^ > 2σ(*F* ^2^)] = 0.022
*wR*(*F* ^2^) = 0.058
*S* = 1.06
5213 reflections
240 parameters
H atoms treated by a mixture of independent and constrained refinement
Δρ_max_ = 1.04 e Å^−3^
Δρ_min_ = −1.41 e Å^−3^

### 

Data collection: *APEX2* (Bruker, 2008 ▶); cell refinement: *SAINT* (Bruker, 2008 ▶); data reduction: *SAINT*; program(s) used to solve structure: *SHELXS97* (Sheldrick, 2008 ▶); program(s) used to refine structure: *SHELXL97* (Sheldrick, 2008 ▶); molecular graphics: *ORTEP-3 for Windows* (Farrugia, 2012 ▶); software used to prepare material for publication: *publCIF* (Westrip, 2010 ▶).

## Supplementary Material

Crystal structure: contains datablock(s) global, I. DOI: 10.1107/S1600536814013336/hb7216sup1.cif

Structure factors: contains datablock(s) I. DOI: 10.1107/S1600536814013336/hb7216Isup2.hkl

Supporting information file. DOI: 10.1107/S1600536814013336/hb7216Isup3.cdx

CCDC reference: 1007160

Additional supporting information:  crystallographic information; 3D view; checkCIF report

## Figures and Tables

**Table 1:** Selected bond lengths (Å)

La1—O2	2.4365 (14)
La1—O4	2.4754 (13)
La1—O5	2.4917 (14)
La1—O1	2.5013 (14)
La1—O6	2.5067 (13)
La1—O3	2.5241 (13)
La1—O7	2.5381 (13)
La1—O8	2.5811 (14)

**Table 2:** Hydrogen-bond geometry (Å, °)

*D*—H⋯*A*	*D*—H	H⋯*A*	*D*⋯*A*	*D*—H⋯*A*
O7—H2*W*⋯O1^i^	0.76 (3)	2.05 (3)	2.7514 (19)	153 (3)
O7—H1*W*⋯O3^i^	0.90 (3)	1.94 (3)	2.7912 (19)	158 (3)
O8—H4*W*⋯O4^ii^	0.75 (3)	2.09 (3)	2.7907 (19)	155 (3)
O8—H3*W*⋯O6^ii^	0.81 (4)	1.96 (4)	2.721 (2)	155 (3)

Symmetry codes: (i) 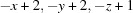; (ii) 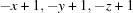.

**Fig. 1.**
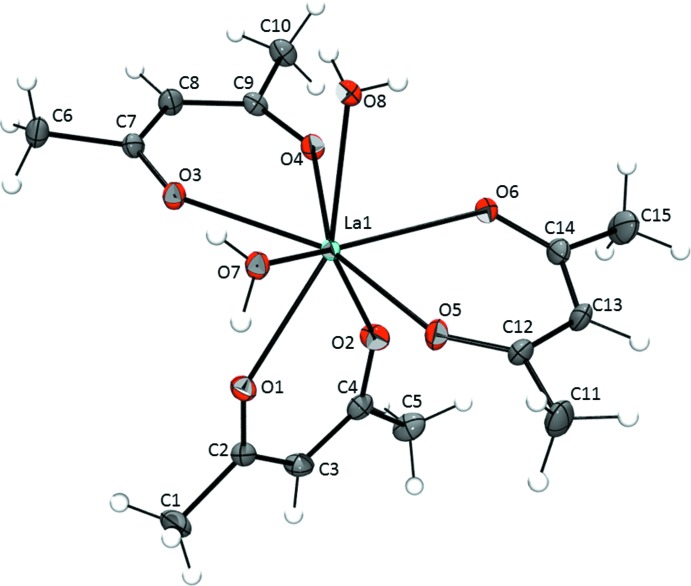
An ORTEP view of the title compound, with displacement ellipsoids drawn at the 50% probability level.

**Fig. 2.**
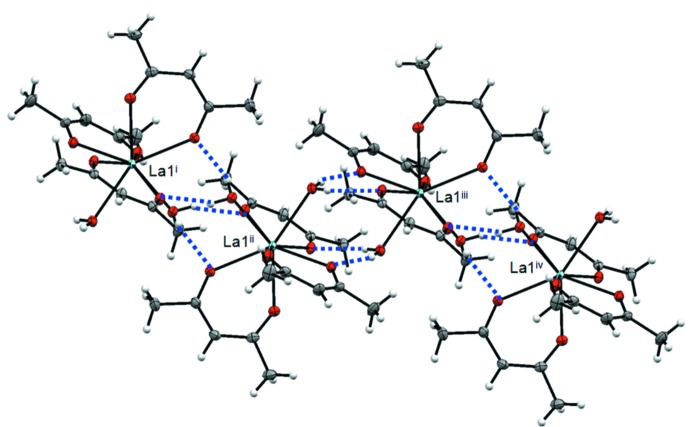
Part of a [110] hydrogen-bonded chain in the title compound. The blue broken lines show the hydrogen bonds. Symmetry codes: (i) 2 - x, 2 - y, 1 - z, (ii) x, y, z, (iii) 1 - x, 1 - y, 1 - z, (iv) x - 1, y - 1, z).

## supplementary crystallographic information

### S1. Comment

Lanthanum (La) is the first element of the lanthanide in the periodic table. Although La does not show any luminescent properties, it has worth investigating as referece complexs of other luminescent lanthanide analogs. Because structural features of La^III^ complexes are similar to those of other lanthanide cases, they can be structurally characterized by nuclear magnetic resonance spectroscopy. La^III^ acetylacetonate complexes are used as a precursor of further functionalized complexes. Herein we redetermined the molecular structure of La(acac)_3_(H_2_O)_2_ (compound I) which has been firstly reported by Phillips *et al.* (1968). In the previous study, all of the oxygen and carbon atoms have been refined isotropically. We have successfully obtained the reliable anisotropic displacement parameters for all non-hydrogen atoms. The molecular geometry of the compound I was almost identical to previous report. The La^III^ is ligated from three acetylacetonate ligands and two aqua ligands which are forming 8-coordinate structure around La^III^ (Figure 1). The average distance of oxygen atoms of acetylacetonate (O1—O6) and La^III^ is 2.489 (30) Å while the original structure showed the average distance of 2.473 (24) Å. The two aqua ligands also align at parpendicular position each other in which O7—La1—O8 angle of 75.20 (5)*^o^*. Similar coordination structures are seen in Ho^III^(acac)_3_(H_2_O)_2_ by Kooijman *et al.* (2000) and Yb^III^(acac)_3_(H_2_O) by Watkins *et al.* (1969). Both complexes have three acac ligands and the former one has nearly identical structure in which two aqua ligands ligate to the central ion and complete 8-coordinated square antiprismatic structure. The longest bond lengths between the central lanthanide ion and the oxygen atoms of acac ligands were observed for the compound I due to difference in their ionic radii. The compound I in the crystal are connected by four hydrogen bonding, O7—O1^i^ (symmetry codes: (i) 2 - *x*, 2 - *y*, 1 - *z*), O7—O3^i^, O8—O4^ii^ (symmetry codes: (ii) 1 - *x*, 1 - *y*, 1 - *z*) and O8—O6^ii^, which are forming a one dimensional hydrogen bonding network (Figure 2) propagating in the [110] direction.

### S2. Experimental

An water suspension (10 ml) of acetylacetone (161.9 mg, 1.62 mmol) and LaCl_3_^.^7H_2_ O (200.0 mg, 0.54 mmol) were stirred under room temperature. Quantitative amount of NaOH (64.7 mg, 1.62 mmol) was added to the suspension. A white precipitate was immediately generated. The precipitate was filtered and recrystallized from CH_2_Cl_2_ and methanol in the presence of small amount of water (*ca*. 3%). Colorless blocks of the title compound were obtained in a few days and mounted on a glass capillary. Yield: 77.9 mg, (31%). Analysis: calculated for C_15_H_25_LaO_8_ ([La(acac)_3_(H_2_O)_2_]): C 38.15, H 5.34%; found: C 37.80, H 5.16%. ESI-TOF-MS (CH_3_OH): *m*/*z* 336.97 (calcd: 337.00 for [M–acac–2H_2_O]^+^).

### S3. Refinement

H atoms except two aqua ligands were placed in geometrically idealized positions and constrained to ride on their parent atoms with *U*_iso_(H) = 1.2*U*_eq_(C—H).

H atoms attached to O7 (H1W and H2W) and O8 (H3W and H4W) were found in a difference Fourier map. Any restraints were not needed for a stable refinement. All hydrogen atoms were included in the structure factor calculation.

### Crystal data

**Table:**

[La(C_5_H_7_O_2_)_3_(H_2_O)_2_]	*Z* = 2
*M**_r_* = 472.26	*F*(000) = 472
Triclinic, *P*1	*D*_x_ = 1.657 Mg m^−^^3^
*a* = 8.9245 (12) Å	Mo *K*α radiation, λ = 0.71073 Å
*b* = 10.6597 (15) Å	Cell parameters from 9940 reflections
*c* = 11.3727 (15) Å	θ = 2.5–30.5°
α = 96.614 (2)°	µ = 2.29 mm^−^^1^
β = 100.601 (2)°	*T* = 100 K
γ = 114.325 (2)°	Block, colourless
*V* = 946.8 (2) Å^3^	0.50 × 0.50 × 0.22 mm

### Data collection

**Table:**

Bruker APEXII CCD diffractometer	5213 independent reflections
Radiation source: fine focus sealed tube	5068 reflections with *I* > 2σ(*I*)
Graphite monochromator	*R*_int_ = 0.030
Detector resolution: 8.3333 pixels mm^-1^	θ_max_ = 29.6°, θ_min_ = 1.9°
phi and ω scans	*h* = −12→12
Absorption correction: multi-scan (*SADABS*; Bruker, 2008)	*k* = −14→14
*T*_min_ = 0.40, *T*_max_ = 0.63	*l* = −15→15
13810 measured reflections	

### Refinement

**Table:**

Refinement on *F*^2^	Secondary atom site location: difference Fourier map
Least-squares matrix: full	Hydrogen site location: inferred from neighbouring sites
*R*[*F*^2^ > 2σ(*F*^2^)] = 0.022	H atoms treated by a mixture of independent and constrained refinement
*wR*(*F*^2^) = 0.058	*w* = 1/[σ^2^(*F*_o_^2^) + (0.0344*P*)^2^ + 0.2713*P*] where *P* = (*F*_o_^2^ + 2*F*_c_^2^)/3
*S* = 1.06	(Δ/σ)_max_ = 0.003
5213 reflections	Δρ_max_ = 1.04 e Å^−^^3^
240 parameters	Δρ_min_ = −1.41 e Å^−^^3^
0 restraints	Extinction correction: *SHELXL97* (Sheldrick, 2008)
Primary atom site location: structure-invariant direct methods	Extinction coefficient: 0.0149 (8)

### Special details

**Table:**

Geometry. All e.s.d.'s (except the e.s.d. in the dihedral angle between two l.s. planes) are estimated using the full covariance matrix. The cell e.s.d.'s are taken into account individually in the estimation of e.s.d.'s in distances, angles and torsion angles; correlations between e.s.d.'s in cell parameters are only used when they are defined by crystal symmetry. An approximate (isotropic) treatment of cell e.s.d.'s is used for estimating e.s.d.'s involving l.s. planes.
Refinement. Refinement of *F*^2^ against ALL reflections. The weighted *R*-factor *wR* and goodness of fit *S* are based on *F*^2^, conventional *R*-factors *R* are based on *F*, with *F* set to zero for negative *F*^2^. The threshold expression of *F*^2^ > σ(*F*^2^) is used only for calculating *R*-factors(gt) *etc*. and is not relevant to the choice of reflections for refinement. *R*-factors based on *F*^2^ are statistically about twice as large as those based on *F*, and *R*- factors based on ALL data will be even larger.

### Fractional atomic coordinates and isotropic or equivalent isotropic displacement parameters (Å^2^)

**Table:**

	*x*	*y*	*z*	*U*_iso_*/*U*_eq_	
La1	0.770906 (10)	0.782106 (9)	0.614919 (8)	0.01085 (5)	
O7	1.00237 (17)	0.85834 (14)	0.50120 (13)	0.0153 (2)	
O6	0.67267 (17)	0.56182 (14)	0.69763 (12)	0.0162 (2)	
O4	0.45916 (16)	0.69885 (14)	0.55190 (12)	0.0159 (2)	
O1	0.95280 (17)	1.03835 (14)	0.70708 (12)	0.0180 (3)	
O2	0.72212 (18)	0.84749 (15)	0.81177 (13)	0.0209 (3)	
O3	0.68870 (16)	0.90080 (14)	0.45213 (12)	0.0161 (2)	
O8	0.66186 (18)	0.58124 (15)	0.42448 (13)	0.0170 (3)	
C3	0.9035 (3)	1.0876 (2)	0.90089 (18)	0.0193 (4)	
H3	0.9367	1.1593	0.9716	0.023*	
C2	0.9814 (2)	1.1236 (2)	0.80637 (17)	0.0170 (3)	
C4	0.7789 (2)	0.9524 (2)	0.89890 (17)	0.0175 (3)	
C5	0.7027 (3)	0.9307 (2)	1.00763 (19)	0.0239 (4)	
H5A	0.6022	0.9485	0.9939	0.036*	
H5B	0.7869	0.9961	1.0817	0.036*	
H5C	0.6697	0.8336	1.0177	0.036*	
C1	1.1092 (3)	1.2747 (2)	0.8198 (2)	0.0281 (4)	
H1A	1.2086	1.2767	0.7937	0.042*	
H1B	1.1445	1.3239	0.9057	0.042*	
H1C	1.0571	1.3216	0.7688	0.042*	
C9	0.3503 (2)	0.71969 (18)	0.47772 (17)	0.0142 (3)	
C7	0.5536 (2)	0.89906 (19)	0.39080 (17)	0.0146 (3)	
C10	0.1669 (2)	0.6312 (2)	0.47366 (19)	0.0188 (4)	
H10A	0.1324	0.5336	0.4336	0.028*	
H10B	0.0959	0.6679	0.4275	0.028*	
H10C	0.1529	0.6344	0.5573	0.028*	
C8	0.3893 (2)	0.8151 (2)	0.40103 (18)	0.0174 (3)	
H8	0.2974	0.824	0.3518	0.021*	
C6	0.5706 (2)	0.9907 (2)	0.29805 (18)	0.0198 (4)	
H6A	0.6826	1.0725	0.3242	0.03*	
H6B	0.4817	1.0229	0.2913	0.03*	
H6C	0.5586	0.9366	0.2183	0.03*	
O5	1.01440 (17)	0.74506 (15)	0.72219 (13)	0.0189 (3)	
C14	0.7368 (3)	0.5254 (2)	0.78893 (17)	0.0179 (4)	
C12	1.0387 (3)	0.6712 (2)	0.79565 (17)	0.0183 (3)	
C13	0.9103 (3)	0.5708 (2)	0.83696 (18)	0.0209 (4)	
H13	0.9441	0.5314	0.9017	0.025*	
C15	0.6127 (3)	0.4243 (3)	0.8472 (2)	0.0299 (5)	
H15A	0.5659	0.4746	0.8951	0.045*	
H15B	0.6714	0.3842	0.9011	0.045*	
H15C	0.52	0.3484	0.7831	0.045*	
C11	1.2197 (3)	0.6933 (3)	0.8430 (2)	0.0279 (4)	
H11A	1.2719	0.6932	0.7743	0.042*	
H11B	1.2194	0.6171	0.8838	0.042*	
H11C	1.285	0.7839	0.9014	0.042*	
H2W	0.985 (4)	0.867 (3)	0.435 (3)	0.035 (8)*	
H1W	1.104 (4)	0.932 (3)	0.536 (3)	0.034 (8)*	
H4W	0.659 (4)	0.514 (3)	0.441 (3)	0.027 (7)*	
H3W	0.569 (4)	0.561 (3)	0.381 (3)	0.040 (9)*	

### Atomic displacement parameters (Å^2^)

**Table:**

	*U*^11^	*U*^22^	*U*^33^	*U*^12^	*U*^13^	*U*^23^
La1	0.01059 (7)	0.00983 (7)	0.01199 (7)	0.00403 (4)	0.00295 (4)	0.00369 (4)
O7	0.0140 (6)	0.0156 (6)	0.0167 (6)	0.0058 (5)	0.0048 (5)	0.0062 (5)
O6	0.0164 (6)	0.0141 (6)	0.0163 (6)	0.0052 (5)	0.0030 (5)	0.0052 (5)
O4	0.0127 (6)	0.0154 (6)	0.0204 (6)	0.0062 (5)	0.0046 (5)	0.0061 (5)
O1	0.0221 (7)	0.0138 (6)	0.0152 (6)	0.0051 (5)	0.0057 (5)	0.0021 (5)
O2	0.0226 (7)	0.0193 (7)	0.0175 (6)	0.0055 (6)	0.0082 (5)	0.0020 (5)
O3	0.0127 (6)	0.0168 (6)	0.0198 (6)	0.0068 (5)	0.0037 (5)	0.0077 (5)
O8	0.0178 (7)	0.0128 (6)	0.0177 (6)	0.0047 (5)	0.0035 (5)	0.0029 (5)
C3	0.0209 (9)	0.0185 (9)	0.0148 (8)	0.0068 (7)	0.0036 (7)	−0.0013 (7)
C2	0.0174 (8)	0.0154 (8)	0.0159 (8)	0.0065 (7)	0.0016 (7)	0.0022 (6)
C4	0.0173 (8)	0.0237 (9)	0.0145 (8)	0.0114 (8)	0.0046 (7)	0.0047 (7)
C5	0.0226 (10)	0.0329 (11)	0.0169 (9)	0.0120 (9)	0.0081 (8)	0.0041 (8)
C1	0.0319 (11)	0.0154 (9)	0.0251 (10)	0.0010 (8)	0.0047 (9)	0.0015 (8)
C9	0.0124 (7)	0.0128 (7)	0.0180 (8)	0.0061 (6)	0.0052 (6)	0.0014 (6)
C7	0.0156 (8)	0.0129 (8)	0.0160 (8)	0.0074 (7)	0.0031 (6)	0.0033 (6)
C10	0.0128 (8)	0.0176 (8)	0.0265 (10)	0.0065 (7)	0.0069 (7)	0.0050 (7)
C8	0.0120 (8)	0.0185 (8)	0.0220 (9)	0.0071 (7)	0.0028 (7)	0.0066 (7)
C6	0.0192 (9)	0.0215 (9)	0.0211 (9)	0.0094 (7)	0.0055 (7)	0.0107 (7)
O5	0.0165 (6)	0.0195 (6)	0.0222 (7)	0.0084 (5)	0.0035 (5)	0.0107 (5)
C14	0.0239 (9)	0.0164 (8)	0.0130 (8)	0.0075 (7)	0.0059 (7)	0.0051 (7)
C12	0.0194 (9)	0.0184 (8)	0.0152 (8)	0.0087 (7)	−0.0011 (7)	0.0037 (7)
C13	0.0226 (9)	0.0220 (9)	0.0152 (8)	0.0081 (8)	−0.0005 (7)	0.0088 (7)
C15	0.0289 (11)	0.0321 (12)	0.0229 (10)	0.0052 (9)	0.0078 (9)	0.0146 (9)
C11	0.0203 (10)	0.0310 (11)	0.0335 (12)	0.0130 (9)	0.0002 (8)	0.0151 (9)

### Geometric parameters (Å, º)

**Table:**

La1—O2	2.4365 (14)	C1—H1B	0.98
La1—O4	2.4754 (13)	C1—H1C	0.98
La1—O5	2.4917 (14)	C9—C8	1.393 (3)
La1—O1	2.5013 (14)	C9—C10	1.504 (2)
La1—O6	2.5067 (13)	C7—C8	1.404 (2)
La1—O3	2.5241 (13)	C7—C6	1.505 (3)
La1—O7	2.5381 (13)	C10—H10A	0.98
La1—O8	2.5811 (14)	C10—H10B	0.98
O7—H2W	0.76 (3)	C10—H10C	0.98
O7—H1W	0.90 (3)	C8—H8	0.95
O6—C14	1.270 (2)	C6—H6A	0.98
O4—C9	1.274 (2)	C6—H6B	0.98
O1—C2	1.278 (2)	C6—H6C	0.98
O2—C4	1.258 (2)	O5—C12	1.261 (2)
O3—C7	1.269 (2)	C14—C13	1.393 (3)
O8—H4W	0.75 (3)	C14—C15	1.509 (3)
O8—H3W	0.81 (4)	C12—C13	1.408 (3)
C3—C2	1.392 (3)	C12—C11	1.514 (3)
C3—C4	1.406 (3)	C13—H13	0.95
C3—H3	0.95	C15—H15A	0.98
C2—C1	1.511 (3)	C15—H15B	0.98
C4—C5	1.513 (3)	C15—H15C	0.98
C5—H5A	0.98	C11—H11A	0.98
C5—H5B	0.98	C11—H11B	0.98
C5—H5C	0.98	C11—H11C	0.98
C1—H1A	0.98		
			
O2—La1—O4	80.43 (5)	H5A—C5—H5C	109.5
O2—La1—O5	89.79 (5)	H5B—C5—H5C	109.5
O4—La1—O5	147.90 (4)	C2—C1—H1A	109.5
O2—La1—O1	68.90 (5)	C2—C1—H1B	109.5
O4—La1—O1	117.77 (4)	H1A—C1—H1B	109.5
O5—La1—O1	86.07 (5)	C2—C1—H1C	109.5
O2—La1—O6	74.35 (5)	H1A—C1—H1C	109.5
O4—La1—O6	79.74 (4)	H1B—C1—H1C	109.5
O5—La1—O6	68.17 (4)	O4—C9—C8	125.02 (16)
O1—La1—O6	134.81 (4)	O4—C9—C10	115.96 (16)
O2—La1—O3	114.29 (5)	C8—C9—C10	119.01 (16)
O4—La1—O3	68.42 (4)	O3—C7—C8	124.70 (17)
O5—La1—O3	142.08 (4)	O3—C7—C6	117.55 (16)
O1—La1—O3	76.92 (4)	C8—C7—C6	117.74 (16)
O6—La1—O3	144.19 (4)	C9—C10—H10A	109.5
O2—La1—O7	139.98 (5)	C9—C10—H10B	109.5
O4—La1—O7	133.31 (5)	H10A—C10—H10B	109.5
O5—La1—O7	70.81 (5)	C9—C10—H10C	109.5
O1—La1—O7	74.97 (4)	H10A—C10—H10C	109.5
O6—La1—O7	124.99 (4)	H10B—C10—H10C	109.5
O3—La1—O7	72.12 (4)	C9—C8—C7	125.12 (17)
O2—La1—O8	143.70 (5)	C9—C8—H8	117.4
O4—La1—O8	74.43 (5)	C7—C8—H8	117.4
O5—La1—O8	97.74 (5)	C7—C6—H6A	109.5
O1—La1—O8	146.72 (4)	C7—C6—H6B	109.5
O6—La1—O8	75.73 (5)	H6A—C6—H6B	109.5
O3—La1—O8	80.19 (5)	C7—C6—H6C	109.5
O7—La1—O8	75.20 (5)	H6A—C6—H6C	109.5
La1—O7—H2W	122 (2)	H6B—C6—H6C	109.5
La1—O7—H1W	120.9 (19)	C12—O5—La1	137.23 (13)
H2W—O7—H1W	103 (3)	O6—C14—C13	124.89 (18)
C14—O6—La1	133.49 (12)	O6—C14—C15	116.30 (19)
C9—O4—La1	139.02 (12)	C13—C14—C15	118.80 (18)
C2—O1—La1	136.92 (12)	O5—C12—C13	124.82 (18)
C4—O2—La1	139.63 (13)	O5—C12—C11	117.21 (18)
C7—O3—La1	137.69 (12)	C13—C12—C11	117.97 (17)
La1—O8—H4W	112 (2)	C14—C13—C12	124.17 (17)
La1—O8—H3W	117 (2)	C14—C13—H13	117.9
H4W—O8—H3W	106 (3)	C12—C13—H13	117.9
C2—C3—C4	124.49 (18)	C14—C15—H15A	109.5
C2—C3—H3	117.8	C14—C15—H15B	109.5
C4—C3—H3	117.8	H15A—C15—H15B	109.5
O1—C2—C3	124.97 (18)	C14—C15—H15C	109.5
O1—C2—C1	116.52 (17)	H15A—C15—H15C	109.5
C3—C2—C1	118.51 (18)	H15B—C15—H15C	109.5
O2—C4—C3	124.92 (18)	C12—C11—H11A	109.5
O2—C4—C5	116.86 (18)	C12—C11—H11B	109.5
C3—C4—C5	118.19 (18)	H11A—C11—H11B	109.5
C4—C5—H5A	109.5	C12—C11—H11C	109.5
C4—C5—H5B	109.5	H11A—C11—H11C	109.5
H5A—C5—H5B	109.5	H11B—C11—H11C	109.5
C4—C5—H5C	109.5		

### Hydrogen-bond geometry (Å, º)

**Table:**

*D*—H···*A*	*D*—H	H···*A*	*D*···*A*	*D*—H···*A*
O7—H2*W*···O1^i^	0.76 (3)	2.05 (3)	2.7514 (19)	153 (3)
O7—H1*W*···O3^i^	0.90 (3)	1.94 (3)	2.7912 (19)	158 (3)
O8—H4*W*···O4^ii^	0.75 (3)	2.09 (3)	2.7907 (19)	155 (3)
O8—H3*W*···O6^ii^	0.81 (4)	1.96 (4)	2.721 (2)	155 (3)

Symmetry codes: (i) −*x*+2, −*y*+2, −*z*+1; (ii) −*x*+1, −*y*+1, −*z*+1.
